# 2-(2-Methyl-5-nitro-1*H*-imidazol-1-yl)ethyl methane­sulfonate

**DOI:** 10.1107/S1600536812034964

**Published:** 2012-08-25

**Authors:** Sammer Yousuf, Aurang Zeb, Farhana Batool, Fatima Z. Basha

**Affiliations:** a H.E.J. Research Institute of Chemistry, International Center for Chemical and Biological Sciences, University of Karachi, Karachi 75270, Pakistan

## Abstract

The asymmetric unit of the title compound, C_7_H_11_N_3_O_5_S, contains two independent mol­ecules with virtually identical conformations. The imidazole rings of both mol­ecules are essentially planar (r.m.s. deviations = 0.0019 and 0.0038 Å), with a dihedral angle 9.25 (19)° between them. The nitro groups are oriented at 4.5 (2) and 6.44 (13)° with respect to the imidazole rings. In the crystal, mol­ecules are linked to form a three-dimensional framework by C—H⋯O and C—H⋯N hydrogen bonds.

## Related literature

For the biological activity of metronidazole, see: Zeb, Malik *et al.* (2012 ▶). For related structures, see: Yousuf *et al.* (2012 ▶); Zeb, Yousuf *et al.* (2012 ▶).
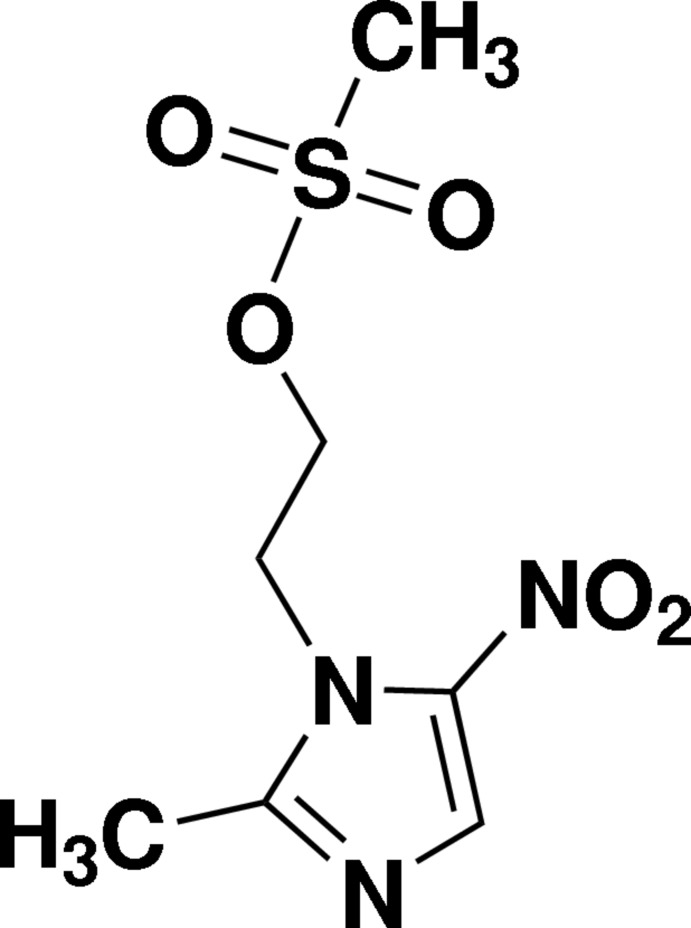


## Experimental

### 

#### Crystal data

C_7_H_11_N_3_O_5_S
*M* *_r_* = 249.26
Triclinic, 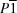

*a* = 8.8547 (17) Å
*b* = 10.927 (2) Å
*c* = 12.033 (2) Å
α = 112.702 (4)°
β = 100.614 (4)°
γ = 90.052 (4)°
*V* = 1052.4 (3) Å^3^
*Z* = 4
Mo *K*α radiation
μ = 0.32 mm^−1^
*T* = 273 K
0.40 × 0.21 × 0.08 mm

#### Data collection

Bruker SMART APEX CCD area-detector diffractometer
Absorption correction: multi-scan (*SADABS*; Bruker, 2000 ▶) *T*_min_ = 0.883, *T*_max_ = 0.975
11649 measured reflections
3918 independent reflections
3078 reflections with *I* > 2σ(*I*)
*R*_int_ = 0.033

#### Refinement

*R*[*F* ^2^ > 2σ(*F* ^2^)] = 0.056
*wR*(*F* ^2^) = 0.152
*S* = 1.07
3918 reflections
291 parameters
H-atom parameters constrained
Δρ_max_ = 0.79 e Å^−3^
Δρ_min_ = −0.32 e Å^−3^

### 

Data collection: *SMART* (Bruker, 2000 ▶); cell refinement: *SAINT* (Bruker, 2000 ▶); data reduction: *SAINT*; program(s) used to solve structure: *SHELXS97* (Sheldrick, 2008 ▶); program(s) used to refine structure: *SHELXL97* (Sheldrick, 2008 ▶); molecular graphics: *SHELXTL* (Sheldrick, 2008 ▶); software used to prepare material for publication: *SHELXTL*, *PARST* (Nardelli, 1995 ▶) and *PLATON* (Spek, 2009 ▶).

## Supplementary Material

Crystal structure: contains datablock(s) global, I. DOI: 10.1107/S1600536812034964/pv2579sup1.cif

Structure factors: contains datablock(s) I. DOI: 10.1107/S1600536812034964/pv2579Isup2.hkl

Supplementary material file. DOI: 10.1107/S1600536812034964/pv2579Isup3.cml

Additional supplementary materials:  crystallographic information; 3D view; checkCIF report

## Figures and Tables

**Table 1:** Hydrogen-bond geometry (Å, °)

*D*—H⋯*A*	*D*—H	H⋯*A*	*D*⋯*A*	*D*—H⋯*A*
C5—H5*B*⋯O6^i^	0.97	2.50	3.235 (4)	133
C5—H5*C*⋯N7^ii^	0.97	2.54	3.484 (4)	165
C7—H7*B*⋯O9^iii^	0.96	2.59	3.498 (4)	157
C7—H7*C*⋯O10^iv^	0.96	2.52	3.423 (4)	157
C12—H12*A*⋯O1^v^	0.97	2.50	3.250 (5)	134
C12—H12*B*⋯N3^vi^	0.97	2.53	3.472 (4)	164
C14—H14*B*⋯O5^vii^	0.96	2.59	3.507 (4)	160
C14—H14*C*⋯O4^viii^	0.96	2.55	3.414 (4)	150

Symmetry codes: (i) 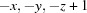; (ii) 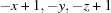; (iii) 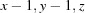; (iv) 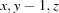; (v) 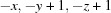; (vi) 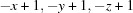; (vii) 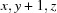; (viii) 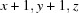.

**Fig. 1.**
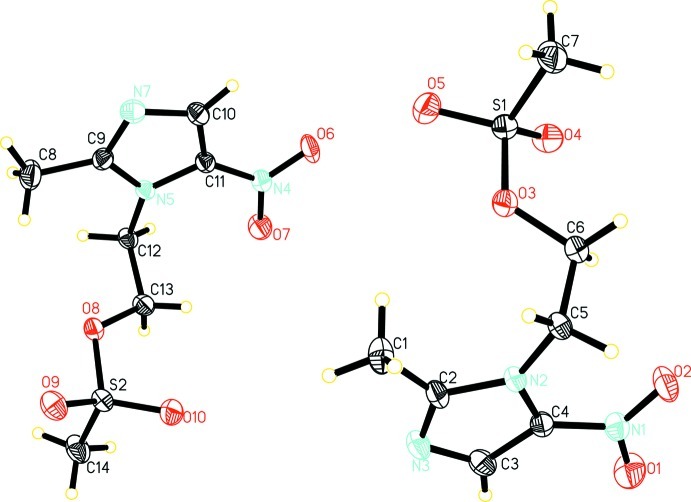
The molecular structure of the title compound with the atom numbering scheme. Displacement ellipsoids are drawn at the 30% probability level. H atoms are presented as small spheres of arbitrary radius.

**Fig. 2.**
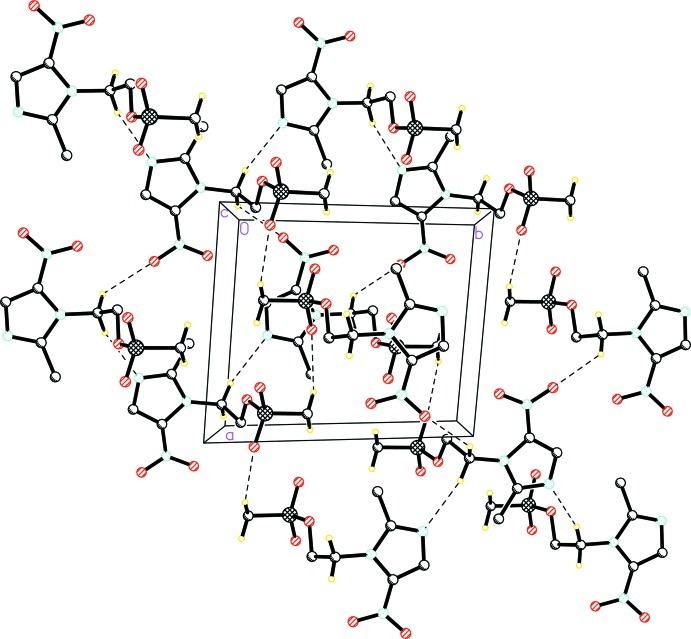
A view of the C—-H···O and C—H···N hydrogen bonds (dotted lines) in the crystal structure of the title compound. H atoms non-participating in hydrogen-bonding were omitted for clarity.

## supplementary crystallographic information

### Comment

Metronidazole (Flagyl) is a well known broad spectrum antibiotic. The structural analogues of metronidazole are reported to have a wide range of biological activities including antibacterial anticancer, antiglycation and *H. pylori* urease inhibitors (Zeb, Malik *et al.*, 2012). The title compound is a methanesulfonate derivative of metronidazole, synthesized as a part of our ongoing reaserch to synthesize and evaluate the antiglycation potential and establish structure activity relationship of the structural analogues of metronidazole.

The title compound contains two independent molecules in an asymmetric unit (Fig. 1) with identical conformations. The two imidazole rings (C2—C4/N2/N3 and C9—C11/N5/N7) are individually planar with r.m.s.d's 0.0038 and 0.0019 Å, respectively; the dihedral angle between the mean planes of the imidazole rings is 9.25 (19)°. The nitro groups N1/O1/O2 and N4/O6/O7 are oriented at 4.5 (2) and 6.44 (13)° with respect to the imidazole rings (C2—C4/N2/N3) and (C9—C11/N5/N7), respectively. The bond distances and angles in both molecules of the title compound agree very well with the corresponding bond distances and angles reported in closely related compounds (Yousuf *et al.*, 2012; Zeb *et al.*, 2012). The crystal packing (Fig. 2) is consolidated by weak intermolecular C—H···O and C—H···N type hydrogen bonds (Table 1).

### Experimental

The title compound was synthesized by adding methane sulfonyl chloride (16 mmol) drop wise into an ice-cooled solution of metronidazole (10 mmol) and triethylamine (16 mmol) in dry dichloromethane (DCM) with continuous stirring. The reaction mixture was further stirred in the ice bath for 4 h. The separated thick material was filtered and washed with water (20 ml *X* 3) to obtain a cream coloured solid which was dissolved and recrystallized from DCM by slow evaporation to give pure crystals of the title compound (82% yield), suitable for single-crystal X-ray diffraction studies. All chemicals were purchased from Sigma–Aldrich.

### Refinement

H atoms on methyl, methylene and methine were positioned geometrically with C—H = 0.96, 0.97 and 0.93 Å respectively, and constrained to ride on their parent atoms with *U*_iso_(H)= 1.2*U*_eq_(CH and CH_2_) and 1.5*U*_eq_(CH_3_). A rotating group model was applied to the methyl groups.

### Crystal data

**Table:**

C_7_H_11_N_3_O_5_S	*Z* = 4
*M**_r_* = 249.26	*F*(000) = 520
Triclinic, *P*1	*D*_x_ = 1.573 Mg m^−^^3^
Hall symbol: -P 1	Mo *K*α radiation, λ = 0.71073 Å
*a* = 8.8547 (17) Å	Cell parameters from 3658 reflections
*b* = 10.927 (2) Å	θ = 2.4–27.9°
*c* = 12.033 (2) Å	µ = 0.32 mm^−^^1^
α = 112.702 (4)°	*T* = 273 K
β = 100.614 (4)°	Plate, colorles
γ = 90.052 (4)°	0.40 × 0.21 × 0.08 mm
*V* = 1052.4 (3) Å^3^	

### Data collection

**Table:**

Bruker SMART APEX CCD area-detector diffractometer	3918 independent reflections
Radiation source: fine-focus sealed tube	3078 reflections with *I* > 2σ(*I*)
Graphite monochromator	*R*_int_ = 0.033
ω scan	θ_max_ = 25.5°, θ_min_ = 1.9°
Absorption correction: multi-scan (*SADABS*; Bruker, 2000)	*h* = −10→10
*T*_min_ = 0.883, *T*_max_ = 0.975	*k* = −13→13
11649 measured reflections	*l* = −14→14

### Refinement

**Table:**

Refinement on *F*^2^	Primary atom site location: structure-invariant direct methods
Least-squares matrix: full	Secondary atom site location: difference Fourier map
*R*[*F*^2^ > 2σ(*F*^2^)] = 0.056	Hydrogen site location: inferred from neighbouring sites
*wR*(*F*^2^) = 0.152	H-atom parameters constrained
*S* = 1.07	*w* = 1/[σ^2^(*F*_o_^2^) + (0.0914*P*)^2^ + 0.2849*P*] where *P* = (*F*_o_^2^ + 2*F*_c_^2^)/3
3918 reflections	(Δ/σ)_max_ < 0.001
291 parameters	Δρ_max_ = 0.79 e Å^−^^3^
0 restraints	Δρ_min_ = −0.32 e Å^−^^3^

### Special details

**Table:**

Geometry. All e.s.d.'s (except the e.s.d. in the dihedral angle between two l.s. planes) are estimated using the full covariance matrix. The cell e.s.d.'s are taken into account individually in the estimation of e.s.d.'s in distances, angles and torsion angles; correlations between e.s.d.'s in cell parameters are only used when they are defined by crystal symmetry. An approximate (isotropic) treatment of cell e.s.d.'s is used for estimating e.s.d.'s involving l.s. planes.
Refinement. Refinement of *F*^2^ against ALL reflections. The weighted *R*-factor *wR* and goodness of fit *S* are based on *F*^2^, conventional *R*-factors *R* are based on *F*, with *F* set to zero for negative *F*^2^. The threshold expression of *F*^2^ > σ(*F*^2^) is used only for calculating *R*-factors(gt) *etc*. and is not relevant to the choice of reflections for refinement. *R*-factors based on *F*^2^ are statistically about twice as large as those based on *F*, and *R*- factors based on ALL data will be even larger.

### Fractional atomic coordinates and isotropic or equivalent isotropic displacement parameters (Å^2^)

**Table:**

	*x*	*y*	*z*	*U*_iso_*/*U*_eq_	
S1	0.09144 (8)	−0.18810 (7)	0.47184 (7)	0.0370 (2)	
S2	0.60576 (8)	0.68767 (7)	0.52683 (7)	0.0356 (2)	
O1	−0.2081 (3)	0.3132 (3)	0.8913 (3)	0.0621 (7)	
O2	−0.1805 (3)	0.1027 (2)	0.8395 (2)	0.0557 (6)	
O3	0.1479 (2)	−0.1063 (2)	0.61360 (19)	0.0384 (5)	
O4	−0.0597 (2)	−0.1572 (2)	0.4331 (2)	0.0475 (6)	
O5	0.2124 (3)	−0.1640 (2)	0.4178 (2)	0.0524 (6)	
O6	0.0876 (2)	0.1903 (2)	0.1110 (2)	0.0526 (6)	
O7	0.1462 (2)	0.4013 (2)	0.1692 (2)	0.0493 (6)	
O8	0.5899 (2)	0.60582 (19)	0.38527 (18)	0.0365 (5)	
O9	0.7537 (2)	0.6633 (2)	0.5805 (2)	0.0498 (6)	
O10	0.4738 (2)	0.6572 (2)	0.5663 (2)	0.0474 (6)	
N1	−0.1298 (3)	0.2166 (3)	0.8633 (2)	0.0398 (6)	
N2	0.1317 (2)	0.1471 (2)	0.8363 (2)	0.0301 (5)	
N3	0.2445 (3)	0.3407 (2)	0.8613 (2)	0.0423 (6)	
N4	0.1824 (3)	0.2858 (2)	0.1409 (2)	0.0348 (6)	
N5	0.4575 (2)	0.3517 (2)	0.1632 (2)	0.0288 (5)	
N7	0.5555 (3)	0.1572 (2)	0.1374 (3)	0.0433 (6)	
C1	0.4070 (4)	0.1507 (4)	0.8164 (3)	0.0507 (8)	
H1A	0.4842	0.2158	0.8222	0.076*	
H1B	0.3896	0.0805	0.7359	0.076*	
H1C	0.4415	0.1144	0.8770	0.076*	
C2	0.2630 (3)	0.2136 (3)	0.8381 (3)	0.0348 (6)	
C3	0.0965 (4)	0.3589 (3)	0.8737 (3)	0.0405 (7)	
H3B	0.0509	0.4390	0.8902	0.049*	
C4	0.0247 (3)	0.2410 (3)	0.8582 (3)	0.0336 (6)	
C5	0.1153 (3)	0.0068 (3)	0.8203 (3)	0.0333 (6)	
H5B	0.0572	0.0000	0.8789	0.040*	
H5C	0.2167	−0.0227	0.8384	0.040*	
C6	0.0360 (3)	−0.0831 (3)	0.6936 (3)	0.0387 (7)	
H6B	−0.0012	−0.1667	0.6934	0.046*	
H6C	−0.0513	−0.0420	0.6649	0.046*	
C7	0.0862 (4)	−0.3532 (3)	0.4587 (4)	0.0547 (9)	
H7A	0.0617	−0.4118	0.3734	0.082*	
H7B	0.0090	−0.3678	0.4997	0.082*	
H7C	0.1850	−0.3704	0.4958	0.082*	
C8	0.7433 (4)	0.3470 (4)	0.1814 (4)	0.0540 (9)	
H8A	0.8153	0.2796	0.1668	0.081*	
H8B	0.7714	0.4106	0.2648	0.081*	
H8C	0.7449	0.3912	0.1264	0.081*	
C9	0.5861 (3)	0.2846 (3)	0.1602 (3)	0.0346 (6)	
C10	0.4009 (3)	0.1407 (3)	0.1252 (3)	0.0391 (7)	
H10B	0.3460	0.0609	0.1085	0.047*	
C11	0.3377 (3)	0.2588 (3)	0.1412 (3)	0.0317 (6)	
C12	0.4499 (3)	0.4921 (3)	0.1791 (3)	0.0317 (6)	
H12A	0.3617	0.4992	0.1214	0.038*	
H12B	0.5418	0.5208	0.1596	0.038*	
C13	0.4371 (3)	0.5828 (3)	0.3059 (3)	0.0362 (7)	
H13A	0.4006	0.6665	0.3060	0.043*	
H13B	0.3644	0.5427	0.3357	0.043*	
C14	0.6070 (4)	0.8524 (3)	0.5396 (3)	0.0522 (9)	
H14A	0.6396	0.9112	0.6243	0.078*	
H14B	0.5051	0.8703	0.5088	0.078*	
H14C	0.6769	0.8662	0.4925	0.078*	

### Atomic displacement parameters (Å^2^)

**Table:**

	*U*^11^	*U*^22^	*U*^33^	*U*^12^	*U*^13^	*U*^23^
S1	0.0370 (4)	0.0336 (4)	0.0394 (4)	0.0035 (3)	0.0099 (3)	0.0123 (3)
S2	0.0336 (4)	0.0329 (4)	0.0374 (4)	−0.0030 (3)	0.0037 (3)	0.0122 (3)
O1	0.0480 (14)	0.0549 (15)	0.090 (2)	0.0220 (12)	0.0258 (13)	0.0309 (14)
O2	0.0407 (12)	0.0415 (13)	0.0877 (19)	−0.0049 (10)	0.0170 (12)	0.0266 (13)
O3	0.0320 (10)	0.0386 (11)	0.0433 (12)	0.0017 (8)	0.0092 (9)	0.0139 (9)
O4	0.0437 (12)	0.0484 (13)	0.0458 (13)	0.0075 (10)	0.0032 (10)	0.0161 (11)
O5	0.0546 (14)	0.0558 (15)	0.0531 (14)	0.0045 (11)	0.0236 (11)	0.0224 (12)
O6	0.0369 (12)	0.0487 (14)	0.0702 (16)	−0.0152 (10)	0.0100 (11)	0.0215 (12)
O7	0.0376 (12)	0.0411 (13)	0.0683 (16)	0.0083 (9)	0.0096 (10)	0.0207 (11)
O8	0.0283 (10)	0.0365 (11)	0.0409 (12)	−0.0017 (8)	0.0047 (8)	0.0120 (9)
O9	0.0403 (12)	0.0544 (14)	0.0508 (14)	−0.0003 (10)	−0.0048 (10)	0.0227 (11)
O10	0.0472 (13)	0.0512 (14)	0.0444 (13)	−0.0052 (10)	0.0135 (10)	0.0176 (11)
N1	0.0328 (13)	0.0408 (15)	0.0479 (16)	0.0019 (11)	0.0074 (11)	0.0198 (12)
N2	0.0279 (11)	0.0279 (12)	0.0355 (13)	−0.0002 (9)	0.0049 (9)	0.0142 (10)
N3	0.0403 (14)	0.0342 (14)	0.0523 (16)	−0.0069 (11)	0.0075 (12)	0.0176 (12)
N4	0.0332 (13)	0.0364 (14)	0.0360 (14)	−0.0010 (10)	0.0057 (10)	0.0160 (11)
N5	0.0296 (12)	0.0243 (11)	0.0321 (12)	0.0001 (9)	0.0057 (9)	0.0110 (10)
N7	0.0451 (15)	0.0314 (14)	0.0537 (17)	0.0077 (11)	0.0133 (12)	0.0154 (12)
C1	0.0341 (16)	0.056 (2)	0.069 (2)	0.0004 (14)	0.0132 (15)	0.0303 (19)
C2	0.0329 (15)	0.0343 (16)	0.0379 (17)	−0.0080 (12)	0.0025 (12)	0.0170 (13)
C3	0.0459 (17)	0.0277 (15)	0.0475 (19)	0.0011 (12)	0.0069 (14)	0.0156 (14)
C4	0.0315 (14)	0.0328 (15)	0.0365 (16)	0.0010 (11)	0.0078 (12)	0.0132 (13)
C5	0.0347 (15)	0.0281 (14)	0.0389 (16)	−0.0020 (11)	0.0060 (12)	0.0157 (12)
C6	0.0365 (15)	0.0334 (16)	0.0438 (18)	−0.0042 (12)	0.0113 (13)	0.0113 (13)
C7	0.052 (2)	0.0371 (18)	0.081 (3)	0.0074 (15)	0.0218 (18)	0.0253 (18)
C8	0.0326 (17)	0.055 (2)	0.077 (3)	0.0052 (15)	0.0153 (16)	0.0269 (19)
C9	0.0335 (15)	0.0315 (15)	0.0407 (17)	0.0053 (11)	0.0124 (12)	0.0141 (13)
C10	0.0417 (17)	0.0291 (15)	0.0478 (18)	−0.0017 (12)	0.0116 (13)	0.0153 (14)
C11	0.0320 (14)	0.0289 (14)	0.0347 (15)	−0.0008 (11)	0.0066 (11)	0.0129 (12)
C12	0.0327 (14)	0.0253 (13)	0.0386 (16)	−0.0001 (11)	0.0055 (12)	0.0148 (12)
C13	0.0318 (14)	0.0305 (15)	0.0418 (17)	0.0038 (11)	0.0030 (12)	0.0112 (13)
C14	0.0438 (18)	0.0342 (17)	0.076 (3)	−0.0022 (14)	0.0064 (17)	0.0221 (17)

### Geometric parameters (Å, º)

**Table:**

S1—O4	1.420 (2)	C1—H1A	0.9600
S1—O5	1.425 (2)	C1—H1B	0.9600
S1—O3	1.571 (2)	C1—H1C	0.9600
S1—C7	1.749 (3)	C3—C4	1.367 (4)
S2—O9	1.423 (2)	C3—H3B	0.9300
S2—O10	1.424 (2)	C5—C6	1.494 (4)
S2—O8	1.567 (2)	C5—H5B	0.9700
S2—C14	1.746 (3)	C5—H5C	0.9700
O1—N1	1.234 (3)	C6—H6B	0.9700
O2—N1	1.228 (3)	C6—H6C	0.9700
O3—C6	1.461 (3)	C7—H7A	0.9600
O6—N4	1.233 (3)	C7—H7B	0.9600
O7—N4	1.233 (3)	C7—H7C	0.9600
O8—C13	1.464 (3)	C8—C9	1.484 (4)
N1—C4	1.410 (4)	C8—H8A	0.9600
N2—C2	1.363 (3)	C8—H8B	0.9600
N2—C4	1.381 (4)	C8—H8C	0.9600
N2—C5	1.473 (3)	C10—C11	1.366 (4)
N3—C2	1.323 (4)	C10—H10B	0.9300
N3—C3	1.352 (4)	C12—C13	1.490 (4)
N4—C11	1.406 (3)	C12—H12A	0.9700
N5—C9	1.353 (3)	C12—H12B	0.9700
N5—C11	1.383 (3)	C13—H13A	0.9700
N5—C12	1.474 (3)	C13—H13B	0.9700
N7—C9	1.328 (4)	C14—H14A	0.9600
N7—C10	1.354 (4)	C14—H14B	0.9600
C1—C2	1.467 (4)	C14—H14C	0.9600
			
O4—S1—O5	118.95 (14)	C6—C5—H5C	109.0
O4—S1—O3	109.90 (12)	H5B—C5—H5C	107.8
O5—S1—O3	104.65 (13)	O3—C6—C5	107.7 (2)
O4—S1—C7	109.32 (15)	O3—C6—H6B	110.2
O5—S1—C7	109.50 (15)	C5—C6—H6B	110.2
O3—S1—C7	103.32 (15)	O3—C6—H6C	110.2
O9—S2—O10	118.88 (14)	C5—C6—H6C	110.2
O9—S2—O8	104.78 (13)	H6B—C6—H6C	108.5
O10—S2—O8	109.83 (12)	S1—C7—H7A	109.5
O9—S2—C14	109.74 (15)	S1—C7—H7B	109.5
O10—S2—C14	109.15 (15)	H7A—C7—H7B	109.5
O8—S2—C14	103.28 (15)	S1—C7—H7C	109.5
C6—O3—S1	118.58 (18)	H7A—C7—H7C	109.5
C13—O8—S2	118.53 (17)	H7B—C7—H7C	109.5
O2—N1—O1	123.1 (3)	C9—C8—H8A	109.5
O2—N1—C4	119.8 (2)	C9—C8—H8B	109.5
O1—N1—C4	117.1 (3)	H8A—C8—H8B	109.5
C2—N2—C4	104.9 (2)	C9—C8—H8C	109.5
C2—N2—C5	125.9 (2)	H8A—C8—H8C	109.5
C4—N2—C5	129.1 (2)	H8B—C8—H8C	109.5
C2—N3—C3	106.4 (2)	N7—C9—N5	112.3 (2)
O7—N4—O6	122.9 (2)	N7—C9—C8	124.0 (3)
O7—N4—C11	119.9 (2)	N5—C9—C8	123.7 (3)
O6—N4—C11	117.3 (2)	N7—C10—C11	109.8 (3)
C9—N5—C11	105.3 (2)	N7—C10—H10B	125.1
C9—N5—C12	126.1 (2)	C11—C10—H10B	125.1
C11—N5—C12	128.5 (2)	C10—C11—N5	106.9 (2)
C9—N7—C10	105.7 (2)	C10—C11—N4	127.9 (3)
C2—C1—H1A	109.5	N5—C11—N4	125.1 (2)
C2—C1—H1B	109.5	N5—C12—C13	113.3 (2)
H1A—C1—H1B	109.5	N5—C12—H12A	108.9
C2—C1—H1C	109.5	C13—C12—H12A	108.9
H1A—C1—H1C	109.5	N5—C12—H12B	108.9
H1B—C1—H1C	109.5	C13—C12—H12B	108.9
N3—C2—N2	112.0 (3)	H12A—C12—H12B	107.7
N3—C2—C1	124.5 (3)	O8—C13—C12	108.1 (2)
N2—C2—C1	123.6 (3)	O8—C13—H13A	110.1
N3—C3—C4	109.2 (3)	C12—C13—H13A	110.1
N3—C3—H3B	125.4	O8—C13—H13B	110.1
C4—C3—H3B	125.4	C12—C13—H13B	110.1
C3—C4—N2	107.5 (2)	H13A—C13—H13B	108.4
C3—C4—N1	127.4 (3)	S2—C14—H14A	109.5
N2—C4—N1	125.1 (2)	S2—C14—H14B	109.5
N2—C5—C6	113.0 (2)	H14A—C14—H14B	109.5
N2—C5—H5B	109.0	S2—C14—H14C	109.5
C6—C5—H5B	109.0	H14A—C14—H14C	109.5
N2—C5—H5C	109.0	H14B—C14—H14C	109.5
			
O4—S1—O3—C6	36.6 (2)	C4—N2—C5—C6	80.8 (3)
O5—S1—O3—C6	165.4 (2)	S1—O3—C6—C5	−174.80 (18)
C7—S1—O3—C6	−79.9 (2)	N2—C5—C6—O3	77.7 (3)
O9—S2—O8—C13	165.3 (2)	C10—N7—C9—N5	−0.1 (3)
O10—S2—O8—C13	36.5 (2)	C10—N7—C9—C8	179.2 (3)
C14—S2—O8—C13	−79.8 (2)	C11—N5—C9—N7	0.4 (3)
C3—N3—C2—N2	−0.8 (3)	C12—N5—C9—N7	−176.3 (3)
C3—N3—C2—C1	179.1 (3)	C11—N5—C9—C8	−179.0 (3)
C4—N2—C2—N3	1.0 (3)	C12—N5—C9—C8	4.4 (5)
C5—N2—C2—N3	−176.3 (3)	C9—N7—C10—C11	−0.2 (3)
C4—N2—C2—C1	−178.8 (3)	N7—C10—C11—N5	0.4 (3)
C5—N2—C2—C1	3.9 (4)	N7—C10—C11—N4	−177.2 (3)
C2—N3—C3—C4	0.2 (3)	C9—N5—C11—C10	−0.5 (3)
N3—C3—C4—N2	0.4 (3)	C12—N5—C11—C10	176.1 (3)
N3—C3—C4—N1	−179.7 (3)	C9—N5—C11—N4	177.3 (3)
C2—N2—C4—C3	−0.9 (3)	C12—N5—C11—N4	−6.1 (4)
C5—N2—C4—C3	176.3 (3)	O7—N4—C11—C10	172.6 (3)
C2—N2—C4—N1	179.3 (3)	O6—N4—C11—C10	−7.8 (4)
C5—N2—C4—N1	−3.5 (5)	O7—N4—C11—N5	−4.7 (4)
O2—N1—C4—C3	175.3 (3)	O6—N4—C11—N5	174.9 (3)
O1—N1—C4—C3	−4.2 (5)	C9—N5—C12—C13	−102.3 (3)
O2—N1—C4—N2	−4.9 (4)	C11—N5—C12—C13	81.8 (3)
O1—N1—C4—N2	175.7 (3)	S2—O8—C13—C12	−174.36 (17)
C2—N2—C5—C6	−102.5 (3)	N5—C12—C13—O8	77.5 (3)

### Hydrogen-bond geometry (Å, º)

**Table:**

*D*—H···*A*	*D*—H	H···*A*	*D*···*A*	*D*—H···*A*
C5—H5*B*···O6^i^	0.97	2.50	3.235 (4)	133
C5—H5*C*···N7^ii^	0.97	2.54	3.484 (4)	165
C7—H7*B*···O9^iii^	0.96	2.59	3.498 (4)	157
C7—H7*C*···O10^iv^	0.96	2.52	3.423 (4)	157
C12—H12*A*···O1^v^	0.97	2.50	3.250 (5)	134
C12—H12*B*···N3^vi^	0.97	2.53	3.472 (4)	164
C14—H14*B*···O5^vii^	0.96	2.59	3.507 (4)	160
C14—H14*C*···O4^viii^	0.96	2.55	3.414 (4)	150

Symmetry codes: (i) −*x*, −*y*, −*z*+1; (ii) −*x*+1, −*y*, −*z*+1; (iii) *x*−1, *y*−1, *z*; (iv) *x*, *y*−1, *z*; (v) −*x*, −*y*+1, −*z*+1; (vi) −*x*+1, −*y*+1, −*z*+1; (vii) *x*, *y*+1, *z*; (viii) *x*+1, *y*+1, *z*.

## References

[bb1] Bruker (2000). *SADABS*, *SMART* and *SAINT* Bruker AXS Inc., Madison, Wisconsin, USA.

[bb2] Nardelli, M. (1995). *J. Appl. Cryst.* **28**, 659.

[bb3] Sheldrick, G. M. (2008). *Acta Cryst.* A**64**, 112–122. 10.1107/S0108767307043930 18156677

[bb4] Spek, A. L. (2009). *Acta Cryst.* D**65**, 148–155. 10.1107/S090744490804362X PMC2631630 19171970

[bb5] Yousuf, S., Zeb, A. & Basha, F. Z. (2012). *Acta Cryst.* E**68**, o952. 10.1107/S1600536812006319 PMC3343933 22590014

[bb6] Zeb, A., Malik, I., Rasheed, S., Choudhary, M. I. & Basha, F. Z. (2012). *Med. Chem.* **8**, 846–852. 10.2174/157340612802084360 22741779

[bb7] Zeb, A., Yousuf, S. & Basha, F. Z. (2012). *Acta Cryst.* E**68**, o1218. 10.1107/S1600536812012688 PMC3344152 22606155

